# Applications of Artificial Intelligence and Radiomics in Molecular Hybrid Imaging and Theragnostics for Neuro-Endocrine Neoplasms (NENs)

**DOI:** 10.3390/life13081647

**Published:** 2023-07-28

**Authors:** Michele Balma, Riccardo Laudicella, Elena Gallio, Sara Gusella, Leda Lorenzon, Simona Peano, Renato P. Costa, Osvaldo Rampado, Mohsen Farsad, Laura Evangelista, Desiree Deandreis, Alberto Papaleo, Virginia Liberini

**Affiliations:** 1 Nuclear Medicine Department, S. Croce e Carle Hospital, 12100 Cuneo, Italy; peano.s@ospedale.cuneo.it (S.P.); papaleo.a@ospedale.cuneo.it (A.P.); liberini.v@ospedale.cuneo.it (V.L.); 2 Unit of Nuclear Medicine, Biomedical Department of Internal and Specialist Medicine, University of Palermo, 90133 Palermo, Italy; riclaudi@hotmail.it (R.L.); renatopatrizio.costa@policlinico.pa.it (R.P.C.); 3 Medical Physics Unit, A.O.U. Città Della Salute E Della Scienza Di Torino, Corso Bramante 88/90, 10126 Torino, Italy; egallio@cittadellasalute.to.it (E.G.); orampado@cittadellasalute.to.it (O.R.); 4 Nuclear Medicine, Central Hospital Bolzano, 39100 Bolzano, Italy; sara.gusella@sabes.it (S.G.); mohsen.farsad@sabes.it (M.F.); 5 Medical Physics Department, Central Bolzano Hospital, 39100 Bolzano, Italy; leda.lorenzon@sabes.it; 6 Department of Biomedical Sciences, Humanitas University, 20089 Milan, Italy; med.evangelista.laura@gmail.com; 7 Department of Nuclear Medicine and Endocrine Oncology, Gustave Roussy and Université Paris Saclay, 94805 Villejuif, France; desiree.deandreis@gustaveroussy.fr

**Keywords:** neuroendocrine tumor, NET, machine learning, nuclear medicine, theragnostics, PET, DOTA PET, radiomics

## Abstract

Nuclear medicine has acquired a crucial role in the management of patients with neuroendocrine neoplasms (NENs) by improving the accuracy of diagnosis and staging as well as their risk stratification and personalized therapies, including radioligand therapies (RLT). Artificial intelligence (AI) and radiomics can enable physicians to further improve the overall efficiency and accuracy of the use of these tools in both diagnostic and therapeutic settings by improving the prediction of the tumor grade, differential diagnosis from other malignancies, assessment of tumor behavior and aggressiveness, and prediction of treatment response. This systematic review aims to describe the state-of-the-art AI and radiomics applications in the molecular imaging of NENs.

## 1. Introduction

Neuroendocrine neoplasms (NENs) comprise a wide variety of heterogeneous tumors, originating from the diffuse neuroendocrine system. These tumors are considered rare, with an incidence of about 3–5 new cases/100,000 inhabitants/year, although new data from the *US Surveillance Epidemiology and End Results Program (SEER)* show an increase in the incidence of the disease of about 520% over the last 32 years (1973–2005), with an annual rate of 5.8% [1]. This increase in incidence can be partially attributed to the introduction of new and/or more sophisticated diagnostic tools, such as single-photon emission computed tomography (SPECT), positron emission tomography (PET) combined with computed tomography (PET/CT), or magnetic resonance imaging (PET/MRI).

Although ubiquitous, these tumors most frequently affect the gastro-entero-pancreatic (GEP) tract (33%) and the bronchopulmonary system (25%). The survival of NET depends on the site and the stage according to the 2022 Tumor, Node, Metastasis (TNM) classification and the World Health Organization (WHO) histopathological classification, which expresses both the morphological appearance of the tumor and its proliferative activity in terms of the number of mitoses and the proliferation index (by assessing the Ki-67 index and thus the disease grading) [2,3]. The current WHO classification of neuroendocrine tumors is given in Table 1. Further prognostic factors are chromogranin A (CgA), synaptophysin, somatostatin receptor (SSTR) expression, the tumor’s spontaneous evolution speed, and the patient’s age [4,5]. Among these, the expression of somatostatin receptors (SSTRs) is one of the most remarkable features of well-differentiated tumors; SSTRs type 1 and type 2 are present in most NENs [6].

In vivo imaging of SSTR expression in well-differentiated NENs (G1 and G2 with low-intermediate levels of the Ki-67 index, <10%) is feasible with both [^111^In]DTPA-octreotide scintigraphy (Octreoscan^®^) SPECT and somatostatin analog PET ([^68^Ga]Ga-DOTANOC, [^68^Ga]Ga-DOTATATE, and [^68^Ga]Ga-DOTATOC) [7,8]. In cases of high levels of the Ki-67 index (>10%), high-grade NET (G3) and NEC, or in cases of [^68^Ga]Ga-DOTA-peptide imaging of SSTR-negative lesions, patients are also candidates for 2-[^18^F]F-fluoro-2-deoxy-D-glucose ([^18^F]FDG) PET/CT, a glucose analog [9,10,11,12]. NENs usually grow slowly with a low rate of glucose metabolism; indeed, [^18^F]FDG PET/CT scans are more likely to detect more aggressive and poorly differentiated NENs, which correlate to worse clinical outcomes [13].

In 2017, Chan et al. [14] proposed a staging protocol by means of [^18^F]FDG and [^68^Ga]Ga-DOTA-peptides PET/CT, resulting in the formulation of a new score, the “NETPET grade”, which could help in the prognostic evaluation of NEN patients and the resulting therapeutic decisions. However, to date, this protocol is hardly applicable in the clinical setting; imaging with multiple radiotracers, although potentially providing the most accurate biological characterization of the disease, is not feasible/reimbursed in all patients and should only be considered in selected cases.

This drawback might be partially solved using new artificial intelligence (AI) approaches to extract data from both [^68^Ga]Ga-DOTA-labelled somatostatin analogs and/or [^18^F]FDG PET/CT images [15,16,17,18,19]. Indeed, radiomics uses bioinformatics and data-characterization algorithms to extract several quantitative characteristics (features) from medical images. These characteristics, known as radiomics features (RFs), may be able to identify disease characteristics that are invisible to the human eye, opening the door to the prospect of quantifying particular tumor characteristics and phenotypes [18,20]. There are a large number of radiomic features, related to morphological properties, the intensity distributions of the image voxels, or to the properties of the image texture. Standardized definitions of principal RFs are provided in the reference manual of the Imaging Biomarker Standardization Initiative (IBSI) [21,22]. According to the EANM/SNMMI guidelines on radiomics in nuclear medicine [23], there are three categories of radiomics-based approaches: hand-crafted radiomics (with explicit extraction of pre-designed radiomics features from the images followed by univariate or multivariate analysis), representation-learning based radiomics (with automatic discovering of features and patterns inherent in the images) and hybrid radiomics (a combination of the two other frameworks), AI comprises different types of algorithms that can perform complex tasks by learning from available data, similar to human intelligence. Under the general category of “AI”, deep learning (DL), reinforcement learning, supervised machine learning, and unsupervised machine learning are all included [24,25,26,27,28].

Nuclear medical imaging provides indispensable information for staging, monitoring, and treatment choice and the application of new radiomic and AI tools could implement the information extracted from PET/CT imaging in each setting. This systematic review aims to summarize the most recent research on radiomics and AI used for molecular imaging of NENs.

## 2. Materials and Methods

**Figure 1:**
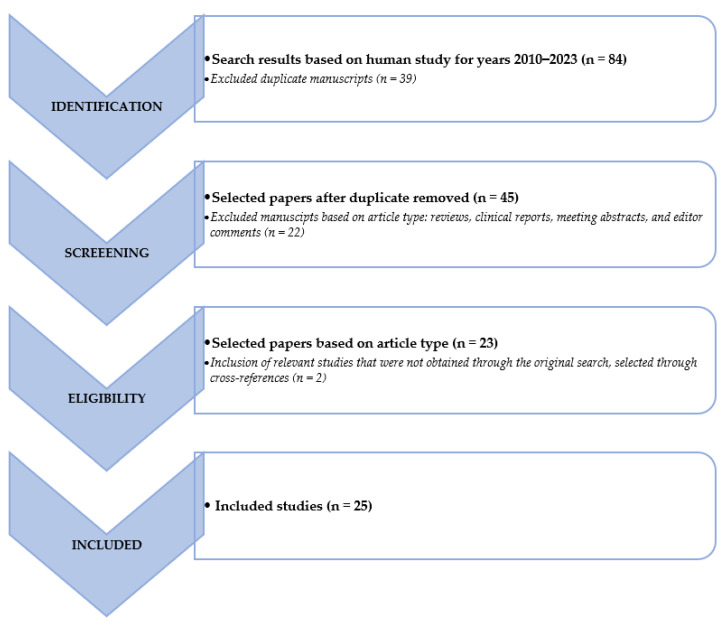
Schematic representation of the performed literature search and the review strategy.

We searched the PubMed, PMC, Scopus, Google Scholar, Embase, Web of Science, and Cochrane library databases (between January 2010 and May 2023), using the following, both as text and as MeSH terms: “neuroendocrine tumor*”, “NET*”, “NEN*”, “artificial intelligence”, “machine learning”, “deep learning”, “convolutional neural network”, “artificial neural network”, “radiomic”, “segmentation”, “PET”, “PET/CT”, “PET/MR”, “octreotide”, “[^68^Ga]Ga-DOTATOC”, “[^68^Ga]Ga-DOTANOC”, “[^68^Ga]Ga-DOTATATE”, “FDG”, “[^18^F]Fluorodeoxyglucose, “DOPA”, “[^177^Lu]Lu”, “[^90^Y]Y”, “diagnosis”, “screening”, “theranostic”, “theragnostic”, “RLT”, “PRRT” and “peptide receptor radionuclide therapy”. No language restriction was applied to the search, but only articles in English were reviewed. The comprehensive literature review produced 84 papers. After removing duplicates in accordance with the Preferred Reporting Items for Systematic Review and Metanalysis (PRISMA) criteria, 25 papers have been taken into consideration, reviewed, examined, and in-depth detailed in accordance with their title and abstract as previously stated [29]. We also looked for more pertinent articles in the articles’ references that were part of the retrieved literature. Following that, articles were divided into clinical and technical applications. Figure 1 provides a graphic illustration of the search and review strategy.

## 3. Clinical Applications

Radiomics and AI could support the key role of nuclear medicine in the non-invasive assessment of staging and restaging of NEN patients.

### 3.1. Staging

As already mentioned, the use of dual PET/CT with [18F]FDG and [68Ga]DOTA-peptides could help to detect intratumor heterogeneity, facilitating the identification of the best target lesions for diagnostic biopsy and histological subtypes, which have a strong correlation with the prognosis of NEN [2,14,30]. The performance of dual PET for the characterization of histological patterns and prognosis of NEN lesions may be further enhanced by radiomics.

In 2017, Giesel et al. [31] published a study on the correlation between SUV_max_ and CT radiomics analysis using lymph node density in the CT component of the PET/CT examination to differentiate malignant from benign lymph nodes. The authors used a sample size of 1,022 lymph nodes extracted from the PET/CT examinations of 148 patients with different tumor types: 327 lymph nodes from 40 patients with lung cancer; 224 lymph nodes from 33 patients with malignant melanoma; 217 lymph nodes from 35 patients with GEP-NET; 254 lymph nodes from 40 patients with prostate cancer. Despite the large heterogeneity of the population evaluated, in terms of pathology and PET radiopharmaceutical analysis ([^18^F]FDG, [^68^Ga]Ga-DOTATOC, and [^68^Ga]Ga-PSMA-11), the study showed that PET-positive lymph nodes had significantly higher CT densities than PET-negative ones, irrespective of the type of cancer, identifying a CT density threshold of 7.5 Hounsfield units to differentiate between malignant and benign infiltration of lymph nodes and 20 Hounsfield units to exclude benign lymph nodes processes.

In 2020, Weber et al. [32], sought to determine whether conventional PET and MRI parameters and RFs derived from simultaneous [^68^Ga]Ga-DOTATOC PET/CT and MRI were related to the proliferative activity of NETs, potentially allowing for a non-invasive tumor grading. The authors evaluated 304 lesions from 100 NET/NECs patients. They showed that differences between G1 and G2 tumors in conventional PET parameters, MRI ADC values, and RFs determined from both modalities were statistically significant. However, the correlation between the aforementioned parameters and Ki-67-index was weak, suggesting that RFs extracted from combined PET/MRI may not be reliably used for accurate non-invasive tumor grading in patients with Ki-67 < 30%. Further insights have been presented by Thuillier et al. [33] who assessed if conventional PET parameters and RFs extracted by [^18^F]FDG PET/CT could differentiate among different histological subtypes (NETs vs NECs) of lung-NENs in forty-four naïve-treatment patients (15 TC, 11 AC, 1 TC or AC, 16 LCNEC and 3 SCLC). Namely, conventional PET parameters resulted to be able to distinguish Lu-NECs from Lu-NETs (SUV_max_ cut-off = 5.16; AUC = 0.91; *p* < 0.001), but not TC from AC. In fact, stratifying TC and AC according to Ki-67 level, SUV_max_ and SUV_mean_ showed a positive correlation with Ki-67, without statistical significance (*p* = 0.05 and 0.07, respectively). Regarding the TNM status, SUV_max_, MTV, and TLG of the primary lesion were significantly associated with N+ status (*p* < 0.05). On the contrary, RFs did not provide additional information.

More recently, Fonti et al. [34] aimed to test the ability of the coefficient of variation (CoV) derived from [^68^Ga]DOTA-peptides PET/CT imaging in the evaluation and quantification of the heterogeneity of SSTR2 expression within 107 tumor lesions (including 35 primary tumors, 32 metastatic lymph nodes, and 40 distant metastases) of 38 NENs patients (25 GEP-NENs, 7 lung-NENs and 6 from other anatomic districts). Among the RFs for the assessment of tumor heterogeneity, CoV is a simple first-order parameter that indicates the percent variability of SUV_mean_ within the tumor volume reflecting the heterogeneity of tracer distribution. Average CoVs were 0.49 ± 0.20 for primary tumors, 0.57 ± 0.26 for lymph node metastases, and 0.44 ± 0.20 for distant metastases. The CoVs of malignant lesions were up to 4-fold higher than those of normal tissues (*p* ≤ 0.0001). Among malignant lesions, the highest CoV was found for bone metastases (0.68 ± 0.20), and it was significantly greater than that of primary lesions (*p* = 0.01) and liver metastases (*p* < 0.0001). The lowest CoV was observed for liver lesions (0.32 ± 0.07), probably because of the high background uptake. On the other hand, no statistically significant differences were found between the SUV_max_ of primary lesions, lymph node metastases and distant metastases, although the SUV_max_ of distant metastases tended to be higher than that of primary lesions (*p* = 0.0573).

Three studies focused on evaluating the role of radiomics parameters extracted by [^68^Ga]DOTA-peptides PET images in predicting histopathological prognostic factors in pancreatic NEN tumors (PanNETs) patients. In 2020, Mapelli et al. [35] retrospectively extracted conventional and tumor burden PET parameters and radiomics parameters (using Chang-Gung Image Texture Analysis software package, version 1.3; digitalization method: 4; digitalization bins: 64) on the primary tumor lesion from both [^18^F]FDG and [^68^Ga]Ga-DOTATOC PET/CT scan images of 61 treatment-naive PanNET patients undergoing surgery. Intensity variability, SZV, homogeneity, SUV_max_ and MTV were predictive for tumor dimension in [^18^F]FDG images. From principal component analysis (PCA), 4 elements were extracted: PC1 correlated with all [^18^F]FDG variables, while PC2, PC3 and PC4 with [^68^Ga]Ga-DOTATOC variables. The only significant predictor of angioinvasion was PC1 (*p* = 0.02), while the only significant predictor of lymph node involvement was PC4 (*p* = 0.015). All principal components except PC4 significantly predicted tumor dimension (*p* < 0.0001 for PC1, *p* = 0.0016 for PC2 and *p* < 0.0001 for PC3). The same group [36] extracted conventional PET and MRI parameters, and radiomics parameters from hybrid [^68^Ga]Ga-DOTATOC PET/MRI of 16 treatment-naive PanNET patients undergoing surgery, using another open-source Python package Pyradiomics 3.0.1 (https://www.radiomics.io/pyradiomics.html; accessed on 24 July 2023). They discovered a moderately significant, inverse connection (rho = 0.58, *p* = 0.02) between SUVmax and LN involvement. SUVmax proved to be a reliable indicator of LN involvement, with an AUC of 0.850 (95% CI: 0.60–1.00), an optimal cut-off value of 90.960, sensitivity of 60%, and specificity of 100%. Potential correlations between radiomics characteristics and tumor grade, LN involvement and vascular invasion were analyzed. After adjustment for multiple comparisons, only second-order radiomics parameters Gray-Level-Variance (GLV) and High-Gray-Level-Zone-Emphasis (HGLZE) extracted from T2 MRI demonstrated significant correlations with LN involvement (adjusted *p* = 0.009), also showing a good predictive performance (AUC = 0.992), with an optimal cut-off value of 0.145 for GLV (correspondent sensitivity and specificity of 90% and 100%, respectively) and of 1.545 for HGLZE (correspondent sensitivity and specificity of 90% and 100%, respectively). Finally, Bevilacqua et al. [37] extracted conventional PET and radiomics parameters from [^68^Ga]Ga-DOTANOC PET/CT imaging of 51 patients with primary G1-G2 treatment-naive PanNET to investigate their ability to predict G1 versus G2 patients. Patients were grouped according to the method of tumor grade assessment: histology on the entire primary excised lesion (HS) or biopsy (BS). Three radiomics models were evaluated: A (trained on HS, validated on BS), B (trained on BS, validated on HS) and C (using cross-validation on the entire dataset). HS group SUV_max_ values did not significantly differ between G1 (36.9 ± 23.5, [6.9–84.8]) and G2 (45.3 ± 28.6, [15.0–95.7]) (*p*-value = 0.60). On the contrary, the grade of the primary lesion was accurately determined when using RFs: the best RF pairs for predicting G2 and G1 were second-order normalized homogeneity and entropy (*p*-value = 0.0002 with AUC = 0.94 (95% CI, 0.74–0.99). Model A had the best performance (test AUC = 0.90, sensitivity = 0.88, specificity = 0.89) whereas Model C had the worst performance (test median AUC = 0.87, sensitivity = 0.83, specificity = 0.82).

In 2022, Noortman et al. [38] investigated the use of [^18^F]FDG-PET/CT radiomics, SUV_max_, and biochemical profile for the identification of the genetic clusters of 40 paragangliomas (PPGLs) patients (13 cluster 1, 18 cluster 2, 9 sporadic). The dataset was split into five equal-sized folds, stratified for the genetic clusters. Each subgroup consecutively served as a test set and the remaining four-fifths of patients served as the training set. The biochemical profile alone was the lowest performing model with an average multiclass AUC of 0.60. The three-factor PET model showed the best classification performance to distinguish cluster 1 from cluster 2 of PPGL (multiclass AUC of 0.88), however comparable to the performance achieved by SUV_max_ alone (multiclass AUC of 0.85), which could therefore be preferred to the radiomics analysis model in a clinical scenario being more handleable. The most important characteristics and results of the above-mentioned studies are summarized in Table 2.

### 3.2. Restaging

Radiomics and AI may emphasize the role of radiological and functional imaging as prognostic biomarkers, especially to identify patients eligible for targeted therapies, such as RLT, and to evaluate their response to such therapies, facilitating patient-tailored treatments [19,39,40,41]. Several studies have already been published in this field in NEN patients.

In 2017, Nogueira et al. [42] developed an artificial neural network (ANN) approach to automatically assess the treatment responses of patients suffering from NENs (34 patients) and Hodgkin lymphoma (29 patients) based on image features extracted from pre- and post-treatment [^18^F]FDG and [^68^Ga]Ga-DOTANOC PET/CT scans, respectively. Cases were divided into four classes of treatment response: negative (malignancy increased), neutral (no response), positive incomplete (malignancy decreased but lesion did not disappear), and positive complete (the lesion disappeared). Four standard ANN architectures were explored: multilayer perceptron (MLP), radial basis function neural network (RBFNN), probabilistic neural network (PNN), and learning vector quantization neural network (LVQNN). After synthetic data generation and PCA-based dimensionality reduction to only two components, the LVQNN assured classification accuracies of 100%, 100%, 96.3%, and 100% regarding the four response-to-treatment classes.

In 2016, Wetz et al. [43] compared the Krenning score, tumor/lesion (T/L) ratio, and asphericity (ASP) between responding and non-responding lesions (total n = 66) segmented on baseline [^111^In]DTPA-octreotide scintigraphy (Octreoscan^®^) SPECT. According to their analysis, a greater ASP level was related to a worse response to RLT. Additionally, ASP outperformed both the Krenning score and the T/L ratio, being the parameter with the greatest AUC (>0.96), at 4 and 12 months of follow-up to distinguish responding from non-responding lesions. In 2020, the same group [44] evaluated the lesional asphericity (ASP), extracted from the pre-therapeutic Octreoscan, as the first imaging-based prognostic marker for progression-free survival (PFS) in 30 GEP-NEN patients that were candidates for therapy with mTOR inhibitor everolimus and with metachronous or progressive liver metastases. Only ASP > 12.9% (hazard ratio, HR), 3.33; *p* = 0.024) and prior RLT (HR, 0.35; *p* = 0.043) resulted as statistically significant in multivariable Cox analysis. Moreover, when the ASP was above 12.9%, the median PFS was 6.7 months (95% CI: 2.1–11.4 months), whereas when it was below 12.9%, it was 14.4 (12.5–16.3) months (log-rank, *p* = 0.028).

Further studies evaluated the application of AI on the assessment of responses to RLT in PET images; the assessment of response to RLT is still challenging, despite the fact that it seems to be one of the most successful treatment choices for metastatic, inoperable, well-differentiated GEP NETs. Particular attention has been paid to the evaluation of RFs capable of describing tumor heterogeneity, which is usually associated with a worse prognosis as a result of more aggressive biological behavior and treatment failure. In 2020, Weber et al. [45] aimed to assess changes in semiquantitative [^68^Ga]Ga-DOTA-TOC PET/MRI parameters, including ADC, after different types of treatment including RLT. Although the study’s sample size was too small to be statistically significant (only nine patients underwent RLT), responding patients showed a significant decrease in lesion volume on ADC maps and a borderline significant decrease in entropy after RLT, even if non-statistically significant.

In two subsequent studies, Werner et al. [46] evaluated the prognostic value of baseline [^68^Ga]Ga-DOTA-SSTa PET/CT RFs before RLT. RF entropy predicted both PFS and overall survival (OS) in a heterogenous cohort of 141 NET patients who were eligible for RLT (cut-off = 6.7, AUC = 0.71, *p* = 0.02), whereas conventional PET parameters did not show significant impacts. In a consecutive study [47] on a smaller, more homogeneous cohort of 31 pan-NET patients (G1/G2), the authors discovered a similar outcome: entropy was a predictor of overall survival (OS) at ROC analysis (cutoff = 6.7, AUC= 0.71, *p* = 0.02). Indeed, higher entropy indicated longer survival (OS = 2.5 years, 17/31, entropy > 6.7), whereas standard PET parameters did not.

In 2020, Önner et al. [48] evaluated tumor heterogeneity using the parameters skewness and kurtosis on pre- and post-treatment [^68^Ga]Ga-DOTATATE PET/CT to assess the therapy responses of 326 lesions (137 lesions responded partially or completely to the treatment; 189 lesions did not respond to treatment, remained stable, or progressed) delineated from PET images of 22 GEP-NET patients treated with 2–6 therapy cycles of [^177^Lu]Lu-DOTATATE. Lesions that did not respond to RLT had significantly higher skewness and kurtosis values than responding lesions (*p* < 0.001 and *p* = 0.004, respectively). However, ROC curves provided a moderate AUC value for skewness and a slightly lower value for kurtosis (0.619 and 0.518, respectively). Moreover, the authors did not compare the RF parameters with conventional PET parameters.

Subsequent studies have better analyzed this aspect, attempting to highlight the possible added value of radiomics parameters compared to conventional ones. In 2021, Ortega et al. [49] aimed to determine whether quantitative PET parameters (mean SUV_max_, ratio tumor to liver/spleen, T/L and T/S ratio, SUV_max_, SUV_mean_, and heterogeneity parameters, such as CoV, kurtosis, and skewness) on baseline [^68^Ga]Ga-DOTATATE PET/CT (bPET) and interim PET (iPET) performed prior to the second RLT cycle were predictive of therapy response and PFS in ninety-one NET patients (71 responders and 20 non-responders). At bPET, higher mean SUV_max_ and mean SUV_max_ (tumor/liver ratio) were predictors of the therapy response (*p* = 0.018 and 0.024, respectively); while higher SUV_max_ and SUV_mean_ and lower kurtosis were predictors of favorable responses (*p* = 0.025, 0.0055, and 0.031, respectively) and correlated with longer PFS. From the multivariable analysis adjusted for age, primary site, and Ki-67, the mean SUV_max_ (*p* = 0.019), SUV_max_ T/L (*p* = 0.018), SUV_max_ T/S (*p* = 0.041), SUV_mean_ liver (*p* = 0.0052), and skewness (*p* = 0.048) remained significant predictors of PFS. On the other hand, iPET parameters were not predictive of PFS, even if iPET was performed only for a subset of patients.

The same year, in a pilot report on two NET patients who experienced discordant responses to RLT (responder vs. non-responder) according to RECIST1.1, Liberini et al. [50] aimed to assess whether both tumor burden and radiomics parameters may have an added value over conventional parameters in predicting RLT response. They found that 28 RFs extracted from pre-therapy [^68^Ga]Ga-DOTATOC PET/CT showed significant differences between the two patients in the Mann–Whitney test (*p* < 0.05), and the modifications of the tumor burden parameter obtained from pre- and post-PRRT PET/CT correlated with the RECIST1.1 response. Moreover, the authors concluded that seven second-order features with poor correlation with SUV_max_ and PET volume, identified by the Pearson correlation matrix, might have a role in defining inter-patient heterogeneity and in the prediction of therapy response.

The prognostic potential of tumor heterogeneity and tracer avidity in NET patients through a radiomics analysis of pre-RLT [^68^Ga]Ga-DOTATATE PET/CT images has also been evaluated by Atkinson et al. [51] in 44 metastatic NET patients (carcinoid, pancreatic, thyroid, head and neck, catecholamine-secreting, and unknown primary NET). Measures of heterogeneity (higher kurtosis, higher entropy, and lower skewness) on coarse texture scale CT and unfiltered PET images predicted shorter PFS (CT coarse kurtosis: *p* = 0.05, PET entropy: *p* = 0.01, PET skewness: *p* = 0.03) and shorter OS (CT coarse kurtosis: *p* = 0.05, PET entropy: *p* = 0.01, PET skewness *p* = 0.02). Multivariate analysis identified that CT-coarse kurtosis (HR = 2.57, 95% CI = 1.22–5.38, *p* = 0.013) independently predicted PFS, while PET-unfiltered skewness (HR = 9.05, 95% CI = 1.19–68.91, *p* = 0.033) independently predicted OS. Conventional PET parameters, such as SUV_max_ and SUV_mean_, showed trends toward predicting outcomes but were not statistically significant.

Finally, in 2022, Laudicella et al. [52] retrospectively analyzed and compared the predictive value of conventional parameters, radiomics, and ∆radiomics parameters in 324 SSTR-2-positive lesions from 38 metastatic well-differentiated GEP-NET patients (nine G1, twenty-seven G2, and two G3) who underwent restaging [^68^Ga]Ga-DOTATOC PET/CT before complete RLT. The disease status for each lesion was determined by [^68^Ga]Ga-DOTATOC PET/CT follow-up using the same scanner for each patient (progression vs. response in terms of stability, decrease, or disappearance). The k-fold approach was used to divide the data into training and validation sets, and discriminant analysis was utilized to create the predictive model. Once again, SUV_max_ could not predict responses to RLT (*p* = 0.49, AUC 0.523), while radiomics parameters proved to be superior to conventional quantitative parameters. From the reduction and selection process, HISTO_Skewness and HISTO_Kurtosis were able to predict the RLT response with AUC, sensitivity, and specificity levels of 0.745, 80.6%, 67.2% and 0.72, 61.2%, 75.9%, respectively. In RLT-responsive lesions, the authors also observed a mean percentage reduction in the asymmetry (skewness) and a more evident increase in the “discrepancy of the considered histogram from the ordinary one” (Kurtosis) than in non-responsive lesions. The most important characteristics and results of the above-mentioned studies are summarized in Table 3.

## 4. Technical Applications

**Figure 2:**
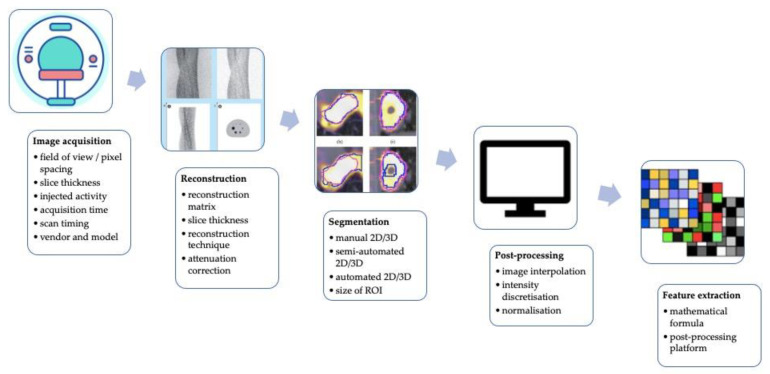
A graphic summary of technical factors affecting the stability of PET radiomics and potentially reducing its robustness, reproducibility, and performance.

Although the scientific interest is rapidly growing, there is the necessity to test and standardize a methodological approach before radiomics and AI applied to PET imaging can potentially be used in a clinical setting [21,23,53]. The technical aspects impacting the stability of PET radiomics and potentially lowering its robustness, repeatability, and performance are graphically summarized in Figure 2.

The word “robustness” is frequently used to describe the repeatability and reproducibility of RFs that have been assessed under various acquisition and processing settings. In the literature, there are several studies evaluating RFs’ robustness in [^18^F]FDG PET/TC imaging, while only a few have yet been published on [^68^Ga]Ga-DOTA-peptide PET/CT imaging.

In 2016, Bailly et al. [54] aimed to evaluate the robustness of RFs extracted from [^68^Ga]Ga-DOTANOC PET images of twenty-six GEP-NET patients, as a function of the acquisition and reconstruction parameters within the context of multi-centric trials. All datasets were reconstructed using four different algorithms, three different matrix sizes, and, for each reconstruction algorithm, three different numbers of iterations. They found that only entropy, energy, RP, and ZP resulted in adequate robustness with respect to the number of iterations, the post-filtering level, the noise in the input data, and the reconstruction algorithm used. In contrast, correlation and LZLGE were found to be very sensitive to the aforementioned parameters. Moreover, the voxel size used to reconstruct the PET images severely impacted the following RFs: Correlation, Energy, Contrast, and Dissimilarity, LGRE, ZLNU, LGZE, and LZLGE RFs. Only entropy and RP had a variability that was comparable to that of SUVmean.

Compared to [^18^F]FDG, [^68^Ga]Ga-DOTA-peptides have a significantly different physiological distribution and showed more inter- and intra-patient heterogeneity for physiological and pathological uptake, requiring the use of different methods of segmentation and different discretization settings. On this premise, in 2021, Liberini et al. [55] sought to identify robust RFs extracted from [^68^Ga]Ga-DOTATOC PET images of forty-nine NET patients, as a result of different segmentation (manual contouring applying three different fixed SUV_max_ thresholds of 20, 30, and 40% respectively, versus a semiautomated edge-based segmentation algorithm, SAEB) and grey-level intensity discretization (an absolute one, namely, AR60 = SUV from 0 to 60, and a relative one, namely, RR = min-max of VOI’s SUV) methods. Of 51 RFs extracted with the open-source IBSI-compliant Lifex software [56], 64.7% (7/10 conventional, 3/6 histogram, 2/4 shape, and 21/31 textual) showed high robustness in terms of consistency (intra-class correlation coefficients—ICC > 0.9) and agreement (low median coefficient of variance, COV_L_) between different operators, improved by applying an SUV_max_ threshold of 40% (86.5%). Furthermore, the dice similarity coefficient (DSC) mean value was 0.75 ± 0.11 (0.45–0.92) between the SAEB and operators and 0.78 ± 0.09 (0.36–0.97) among the four manual segmentations, suggesting that SAEB segmentation could be an optimal alternative to manual segmentation, even if further validations are needed. Finally, the use of the absolute intensity scaling factor (AR60) achieved greater robustness of RF in segmentation than the relative intensity scaling factor (RR).

Although RF robustness is high using manual contouring with the 40% fixed SUV_max_ thresholds, this approach is time-consuming; moreover, it may lead to the loss of important biological information and a reduction of analyzable lesions with textural characteristics due to the low number of voxels not being sufficient to perform a radiomics analysis. For that reason, various semiautomatic and automatic segmentation methods were evaluated for both single-lesion and total tumor-burden segmentation [49,57,58,59,60,61,62,63,64]. A 2D fully convolutional U-Net-like neural network was used in 2021 by Wehrend et al. [65] to automatically identify liver lesions by [^68^Ga]Ga-DOTATE PET/CT. The neural network was trained by minimizing a linear combination of binary cross-entropy loss and dice loss with a stochastic gradient descent algorithm for 100,000 iterations. The model was performed on 125 patients (57 with PET-positive liver lesions and 68 without) randomly divided into 75 patients for the training set (36 abnormal, 39 normal), 25 for the validation set (11 abnormal, 14 normal), and 25 for the test set (11 abnormal, 14 normal). In this study, manual liver segmentation and semiautomated annotation of lesions were used as the ground truth; the authors used a modified PERCIST threshold for liver lesion identification and a commercially available gradient edge detection tool (PET Edge plus; MIM 7.0.3 software) to define the lesion boundaries. A total of 233 lesions were annotated, with each abnormal study containing a mean of 4 ± 2.75 lesions. To reduce the effects of noisy predictions, filters based on pixel area were applied to remove values below a certain threshold; the highest mean positive predictive value (PPV) of 0.94 ± 0.01 and the highest mean F1 score of 0.79 ± 0.01 were produced with a 20-pixel filter, while the highest mean area under the precision–recall curve (PR-AUC) of 0.73 ± 0.03 was produced with a 15-pixel filter. The most common sources of error reported for false-positive lesions were lesions that were close to the edge of a true lesion, with a score above the PERCIST limit, but disagreed with the annotated gold standard (6/12; 50%); while false-negative lesions were lesions with low uptake.

In 2022, Carlsen et al. [66] investigated the performance of an AI network with deep learning U-Net architecture for the tumor segmentation of [^64^Cu]Cu-DOTATATE PET/CT images of GEP-NEN and lung-NEN patients (117 in training, 41 in testing, and 10 patients without signs of NEN included as negative controls). Ground truth segmentations were obtained by a standardized semiautomatic method for tumor segmentation. Although the ensemble model obtained rather high values of the lesion-wise dice index, pixel-wise dice index, precision, and sensitivity (0.85, 0.8, 0.79, and 0.87, respectively), some degree of manual adjustment was required in 85% (35/41) of patients to consider the performance of the model as acceptable. Nevertheless, the AI model appeared to be faster than the ground truth (semiautomatic) method, reducing the manual adjustment time to 5 min (vs. 17 min, *p* < 0.01). Among the limitations of the evaluated AI model, the authors reported:—The need to manually remove false-positive segmentations (such as adrenal gland or bladder) and/or to add lesions not included in the segmentation;—The elimination of all lesions smaller than 0.1 mL (lesions < 9 voxels) to reduce segmentation of noise;—A non-homogeneous training and testing cohort due to a higher frequency of primary small bowel and pancreatic tumors and grade-2 patients (although the Ki-67 index was not significantly different between patients). 

Another interesting application of AI is the automatic segmentation of healthy organs and tumor lesions for patient-specific dosimetry in radiopharmaceutical treatment (RPT) for the development and validation of individualized protocols to provide personalized therapy. In 2021, Khan et al. [67] developed a convolution neural network (CNN) for automated kidney segmentation that accurately aligns the CT-segmented volume of interest (VOI) to the kidneys in SPECT images, trained with SPECT/CT images performed over the abdominal area of 137 patients treated with RLT. The Bland–Altman analysis demonstrated higher accuracy for the CNN segmentation compared to the manual-segmented kidneys without VOI adjustment, with the benefit of a reduced operating time. Similarly, in 2022, Dewaraja et al. [68] aimed to construct and test an integrated voxel-level pipeline (CNNs) that automates key components (organ segmentation, registration, dose-rate estimation, and curve fitting) on CT of SPECT/CT of the RPT dosimetry process and then use it to report patient-specific dosimetry in 20 patients that are candidates for RLT. Indeed, the organs on CT of SPECT/CT are automatically segmented using CNNs; then, the VOI propagation to other time-point is achieved by local contour intensity-based SPECT-SPECT alignment. Dose-rate estimation is performed by explicit Monte Carlo (MC) using the fast, Dose-Planning Method code, and the best function for dose-rate fitting is automatically chosen for each voxel. CNN-defined kidneys resulted in high Dice index values (0.91–0.94) and only small differences (2–5%) in the mean dose when compared with manual segmentation. For a typical patient, the time for the entire process was ~25 min on a desktop computer, including the time for CNN organ segmentation, co-registration, MC dosimetry, and voxel curve fitting.

Finally, Ding et al. [69] examined the effectiveness of using a machine learning-based algorithm for a rapid dual-tracer ([^18^F]FDG and [^68^Ga]Ga-DOTATATE) simulated dynamic PET acquisition protocol in a single imaging session with NET imaging data of twelve NET patients based on dynamic acquisitions performed separately on two different days. Authors developed a recurrent extreme gradient boosting (rXGBoost) machine learning algorithm to separate the mixed [^18^F]FDG and [^68^Ga]Ga-DOTATATE time activity curves (TACs) for the region of interest (ROI)-based quantification with tracer kinetic modeling. The results of this preliminary analysis were quite encouraging:—The correlation coefficient of the proposed method was higher than that of the parallel multi-tracer compartment model (PMCM);—The algorithm can effectively reduce the total scanning time by shortening the stagger time of the two tracers;—High accuracy was still maintained when the simulated delay between the two tracers’ injection was only 5 min.

## 5. Discussion

This review highlights the state-of-the-art AI in the scenario of NETs in nuclear medicine, revealing how several reasons make this field still in its preliminary state.

NENs are relatively rare and extremely heterogeneous tumors, which makes it difficult to have a large and adequately homogeneous sample of patients.

Imaging analysis procedures such as tumor segmentation methods, grey-level intensity discretization, and image reconstruction algorithms can affect the robustness, repeatability, and reproducibility of radiomics variables and their results; while the more widespread use of [^18^F]FDG PET/CT in clinical practice as a metabolic tracer has led to a significantly larger evaluation of the robustness of radiomics studies, considerably fewer studies have evaluated this aspect in [^68^Ga]DOTA-peptide PET/CT imaging.

In clinical studies, authors examined the potential of radiomics for several key objectives in the management of NEN patients: prediction of tumor grade, prognostic assessment, and prediction of response to RLT. Despite the attractive results reported above, there is still considerable work required to apply the results of this research in clinical practice; a prospective, sufficiently large and homogeneous study sample is hardly available due to the rarity of NENs. Furthermore, NEN patients barely perform dual-imaging evaluation with [^18^F]FDG and [^68^Ga]Ga-DOTA-peptides PET to evaluate the “NETPET score”, and the inherent heterogeneity of these tumors makes a standardized approach in the methodology applied to PET imaging difficult compared to that for other tumors (high variability of uptake for both [^18^F]FDG and [^68^Ga]Ga-DOTA-peptides PET imaging compared to other tumors). [^68^Ga]Ga-DOTA-peptide PET imaging is considered the state-of-the-art approach to quantify SST receptors in vivo, while [^18^F]FDG is used to metabolically characterize more aggressive and higher-grade NET lesions; consequently, it may be difficult to identify and segment the most aggressive lesions on only [^68^Ga]Ga-DOTA-peptide images, especially for lesions with low SST receptor expression (low SUV_max_ values). Moreover, the majority of the described studies are exploratory with univariable analyses, lacking external validation. In addition, many studies were conducted before the drafting of the IBSI, which aims to provide image biomarker nomenclature and definitions, and before the new European Association of Nuclear Medicine (EANM) and the Society of Nuclear Medicine and Molecular Imaging (SNMMI) guidelines for pertinent study design, quality control, data collection, the effects of acquisition and reconstruction, detection and segmentation, standardization, and feature implementation, as well as adequate modeling schemes, model assessment, and interpretation [17,21,22,23].

Nonetheless, some encouraging results obtained through the above-mentioned articles suggest that a significant role in ‘non-invasive’ patient management in the clinical practice and in the clinical decision support system (CDSS) could be played by some RFs in the future, such as first-order statistics, metabolic tumor burden (MTB) from [^18^F]FDG PET/CT images, somatostatin receptor tumor burden (SRTB) from [^68^Ga]Ga-DOTA-peptide PET/CT images, and GLRLM features. Moreover, the publication of negative results in the radiomics field is also very relevant to understand the directions for meaningful future research. Based on the presented results, technical and clinical studies that used radiomics for the prediction of response or long-term outcome seem to be more relevant endpoints compared with studies focused on the identification of the tumor grade, since these latter presumably require even more large and homogeneous samples and the availability of several data (histopathological, genetic, radiological, and nuclear medical).

## 6. Conclusions

Clinically and physiologically, NENs range from quite indolent to extremely aggressive neoplasms. Both the staging and the histological grading affect their prognosis and course of treatment since less-differentiated NENs are more likely to be aggressive and have a poor prognosis than well-differentiated tumors. The scientific community is giving increasing attention to AI techniques due to a variety of potential applications in the NEN field, ranging from the technical aspects of image reconstruction and segmentation to clinical and therapeutic aspects. However, a large workload and numerous validation and rigorous studies following new radiomics and AI guidelines are still required to implement most of these strategies in clinical practice.

## Figures and Tables

**Table 1:** The World Health Organization (WHO) 2022 classification of neuroendocrine neoplasms.

	Lung and Thymus	Mitotic Index	Necrosis	Other Features	Gastro-intestinal (GI) Tract and Hepato-Pancreato-Biliary Organs	Mitotic INDEX	Ki67 Index	Other Features	Upper Aerodigestive Tract and Salivary Glands	Mitotic Index	Ki67 Index	Other Features	Thyroid	Mitotic Index	Ki67 Index	Necrosis
Well-differentiated *	NET, TC	<2/10HPF	No		NET, G1	<2/10HPF	<3%		NET. G1	<2/10HPF	<20%		Low grade MTC	<5/10HPF	<5%	No
	NET, AC	2-10/10HPF	Yes (punctate)		NET, G2	2–20/10HPF	3–20%		NET. G2	2–10/10HPF	<20%					
	Carcinoids/NETs	>10/10HPF	Yes	and/or Ki67 index (>30%)	NET, G3	>20/10HPF	>20%		NET, G3	>10/10HPF	>20%					
Poorly differentiated *	NEC, SCLC	>10/10HPF	Yes	small cell cytomorphology	NEC, SCNEC	>20/10HPF	>20% (often >70%)	small cell cytomorphology	NEC, SCNEC	>20/10HPF	>20% (often >70%)	small cell cytomorphology	High grade MTC	>/10HPF	>5%	Yes
	NEC, LCNEC	>10/10HPF	Yes	large cell cytomorphology	NEC, LCNEC	>20/10HPF	>20% (often >70%)	large cell cytomorphology	NEC, LCNEC	>20/10HPF	>20% (often >55%)	large cell cytomorphology				
Mixed neoplasms	MiNENs	NA	>30%		MiNENs	NA	>30%		MiNENs	NA	>30%					

NOTE: AC, atypical carcinoid; HPF, high-power field; LCNEC, large cell neuroendocrine carcinoma; MiNEN, mixed neuroendocrine/non-neuroendocrine neoplasm; MTC, medullary thyroid carcinomas; NEC, neuroendocrine carcinoma; NET, neuroendocrine tumor; SCLC, small-cell lung carcinoma; SCNEC, small cell neuroendocrine carcinoma; TC, typical carcinoid; * Morphologically well-differentiated or poorly differentiated.

**Table 2:** Published articles concerning the implementation of radiomic and/or AI tools in the staging of NEN patients.

Author	Year of Publication	Study Design	NET Type	Number of Patients	Source of Data	Software	AI Application	Validation	Aim of the Study	Findings
Giesel et al. [31]	2017	retrospective	GEP-NET	35	[^68^Ga]DOTA-peptides PET/CT	software developed at the Fraunhofer Institute for Medical Image Computing	no	no	malignant versus benign lesions	PET-positive lymph nodes had significantly higher CT densities than PET-negative ones, irrespective of the type of cancer
Weber et al. [32]	2020	retrospective	all NENs	100	[^68^Ga]DOTA-peptides PET/MRI	LIFEx	no	no	tumor grading	the correlation between imaging parameters (conventional PET parameters, ADC values from MRI, and RFs parameters) and Ki-67-index was weak
Thuillier et al. [33]	2020	retrospective	Lung-NET	44	[^18^F]FDG PET/CT	LIFEx	no	no	tumor grading	conventional PET parameters were able to distinguish Lu-NECs from Lu-NETs but not TC from AC. On the contrary, RFs did not provide additional information
Fonti et al. [34]	2022	retrospective	all NENs	38	[^68^Ga]DOTA-peptides PET/CT	LIFEx	no	no	malignant versus benign lesions	the CoVs of malignant lesions were up to 4-fold higher than those of normal tissues (*p* ≤ 0.0001)
Mapelli et al. [35]	2020	retrospective	Pan-NENs	61	[^68^Ga]DOTA-peptides and [^18^F]FDG PET/CT	Chang-Gung Image Texture Analysis software package	no	no	predictive value of tumor aggressiveness	intensity variability, SZV, homogeneity, SUV_max_ and MTV were predictive for tumor dimension in [^18^F]FDG images; all principal components except PC4 significantly predicted tumor dimension (*p* < 0.0001 for PC1, *p* = 0.0016 for PC2, and *p* < 0.0001 for PC3)
Mapelli et al. [36]	2022	retrospective	Pan-NENs	16	[^68^Ga]DOTA-peptides PET/MRI	Python package Pyradiomics 3.0.1	no	no	predictive value of tumor aggressiveness	a significant inverse correlation between SUV_max_ and LN involvement (rho = −0.58, *p* = 0.02). Only second-order GLV and HGLZE extracted from T2 MRI demonstrated significant correlations with LN involvement (adjusted *p* = 0.009)
Bevilacqua et al. [37]	2021	retrospective	Pan-NENs	51	[^68^Ga]DOTA-peptides PET/TC	ImageJ and MATLAB^®^	no	yes	tumor grading	SUV_max_ values did not significantly differ between G1 and G2 (*p*-value = 0.60). On the contrary, the primary lesion’s grade was correctly identified when using RFs, second-order normalized homogeneity, and entropy (*p*-value = 0.0002 with AUC = 0.94)
Noortman et al. [38]	2022	retrospective	PPGLs	40	[^18^F]FDG-PET/CT	Python package Pyradiomics 3.0.1	no	yes		although comparable to the performance produced by SUVmax alone (multiclass AUC = 0.85), the three-factor PET model demonstrated the best classification performance to separate cluster 1 from cluster 2 of PPG

**Table 3:** Published articles concerning the implementation of radiomic and/or AI tools in the restaging of NEN patients.

Author	Year of Publication	Study Design	NET Type	Number of Patients	Source of Data	Software	AI Application	Validation	Aim of the Study	Findings
Nogueira et al. [42]	2017	retrospective	NENs	34	[^18^F]FDG and [^68^Ga]DOTA-peptides PET/CT	NA	yes	no	predictive value of response to treatment	LVQNN assured classification accuracies of 100%, 100%, 96.3%, and 100% regarding the 4 response-to-treatment classes (negative, neutral, positive incomplete, and positive complete)
Wetz et al. [43]	2016	retrospective	GEP-NENs	20	[^111^In]DTPA-octreotide scintigraphy	ROVER version 2.1.20 (ABX, Radeberg, Germany)	no	no	predictive value of response to treatment	a higher ASP level was associated with poorer response to RLT
Wetz et al. [44]	2020	retrospective	GEP-NENs	30	[^111^In]DTPA-octreotide scintigraphy	ROVER version 2.1.20 (ABX, Radeberg, Germany)	no	no	predictive value of response to treatment	ASP > 12.9% (*p* = 0.024) predicted response to everolimus
Weber et al. [45]	2020	retrospective	all NENs	18	[^68^Ga]DOTA-peptides PET/MRI	LIFEx	no	no	predictive value of response to treatment	even if not statistically significant, PRRT-responding patients displayed a substantial decrease in lesion volume on ADC maps and a borderline significant decrease in entropy after RLT
Werner et al. [46]	2017	retrospective	all NENs (108 GEP-NET)	141	[^68^Ga]DOTA-peptides PET/CT	Interview Fusion Workstation (Mediso Medical Imaging Systems Ltd., Budapest, Hungary)	no	no	predictive value of PFS and OS	RF entropy predicted both PFS and OS (cut-off = 6.7, AUC = 0.71, *p* = 0.02), while conventional PET parameters failed to predict patient outcome
Werner et al. [47]	2019	retrospective	Pan-NET	31	[^68^Ga]DOTA-peptides PET/CT	Interview Fusion Workstation (Mediso Medical Imaging Systems Ltd., Budapest, Hungary)	no	no	predictive value of PFS and OS	entropy was predictive for OS (cutoff = 6.7, AUC = 0.71, *p*= 0.02); indeed, an increased entropy predicted longer survival (entropy > 6.7, OS = 2.5 years, 17/31), while conventional PET parameters failed to predict patient outcome
Önner et al. [48]	2020	retrospective	GEP-NET	22	[^68^Ga]DOTA-peptides PET/CT	LIFEx	no	no	predictive value of response to treatment	the skewness and kurtosis values of the lesions which did not respond to RLT were significantly higher than those with a response (*p* < 0.001 and *p* = 0.004, respectively).
Ortega et al. [49]	2021	retrospective	All NENs	91	[^68^Ga]DOTA-peptides PET/CT	nuclear medicine PACS system with fusion software (Mirada Medical)	no	no	predictive value of PFS and OS	at baseline-PET, from the multivariable analysis, mean SUV_max_ (*p* = 0.019), SUV_max_ T/L (*p* = 0.018), SUV_max_ T/S (*p* = 0.041), SUV_mean_ Liver (*p* = 0.0052) and skewness (*p* = 0.048) were significant predictors of PFS after RLT. On the other hand, interim-PET parameters failed to predict patient outcome
Liberini et al. [50]	2021	retrospective	GEP-NEC	2	[^68^Ga]DOTA-peptides PET/CT	LIFEx	no	no	predictive value of response to treatment	28 RFs extracted from pre-therapy PET/CT showed significant differences between the two patients in the Mann–Whitney test (*p* < 0.05) and the modifications of tumor burden parameter obtained from pre- and post-PRRT PET/CT correlated with RECIST1.1 response
Atkinson et al. [51]	2021	retrospective	All NENs	44	[^68^Ga]DOTA-peptides PET/CT	TexRAD research software (TexRAD, part of Feedback Medical Ltd., www.fbkmed.com, Cambridge, UK)	no	no	predictive value of PFS and OS	multivariate analysis identified that CT-coarse kurtosis (HR = 2.57, 95% CI = 1.22–5.38, *p* = 0.013) independently predicted PFS, while PET-unfiltered skewness (HR = 9.05, 95% CI = 1.19–68.91, *p* = 0.033) independently predicted OS
Laudicella et al. [52]	2022	retrospective	GEP-NET	38	[^68^Ga]DOTA-peptides PET/CT	LIFEx	yes	yes	predictive value of response to treatment	SUV_max_ could not predict response to RLT (*p* = 0.49, AUC 0.523), while HISTO_Skewness and HISTO_Kurtosis were able to predict RLT response with AUC, sensitivity, and specificity of 0.745, 80.6%, 67.2% and 0.72, 61.2%, 75.9%, respectively

## Data Availability

Not applicable.
